# Restructuring the Ikeda City school urinary screening system: report of a screening survey

**DOI:** 10.1186/1447-056X-12-6

**Published:** 2013-12-13

**Authors:** Nobuyuki Kajiwara, Kazuyuki Hayashi, Takayuki Fukui, Satoko Yamamoto, Kensuke Senzaki, Shinichiro Murakami, Takuya Kitamura, Takato Ueoka, Mikito Inoue, Shigeki Hayashi, Keiko Sakamoto, Maiko Yoshimoto, Seiko Asano, Ichiro Maki

**Affiliations:** 1 Department of Nephrology, Ikeda City Hospital, Ikeda, Osaka, Japan; 2 Department of Nephrology and Artificial Kidney, Matsushita Memorial Hospital, Moriguchi, Osaka, Japan; 3 Department of General Medicine, Ikeda City Hospital, Ikeda, Osaka, Japan; 4 Inoue Clinic Medical Corporation, Ikeda, Osaka, Japan; 5 Ikeda City Board of Education, Ikeda, Osaka, Japan; 6 Ikeda City Ishibashi Elementary School, Ikeda, Osaka, Japan; 7 Ikeda City Fushiodai Elementary School, Ikeda, Osaka, Japan; 8 Department of Pediatrics, Ikeda City Hospital, Ikeda, Osaka, Japan

**Keywords:** Chronic glomerulonephritis, Chronic nephritic syndrome, Prevalence, Renal biopsy, School urinary screening, Urinary protein/creatinine ratio

## Abstract

**Background:**

Annual urinary screening is conducted at municipal kindergartens, elementary schools, and junior high schools in Ikeda City, Osaka, Japan (Ikeda City School System), and the results are reviewed by a general physician, but standards for when to recommend specialist referral have not been clear.

**Methods:**

In all children attending the Ikeda City School System in 2012, dipstick urinalysis of a first-morning urine specimen was recommended once or twice, and if a second urinalysis showed proteinuria (≥1+), the urinary protein/creatinine ratio was measured. If this showed ≥0.2 g/g of creatinine (g/gCr), it was recommended that the child be evaluated by a specialist at Ikeda City Hospital.

**Results:**

Urinary screening was performed in about 20% (388) of kindergarten, about 90% (5363) of elementary school, and about 86% (2523) of junior high school children living in Ikeda City. Urine samples were obtained from 387, 5349, and 2476 children, respectively. The urinary protein/creatinine ratio was ≥0.2 g/gCr in 13 children, including 1 elementary and 12 junior high children. In these 13 children, chronic nephritic syndrome (CNS) was suspected in 6 junior high school children, and of these, this was a new finding in 5, and renal biopsy was indicated in 3. In Ikeda City, the prevalence of CNS in elementary school children was <0.03%, the prevalence of CNS in junior high school children was 0.29%, and a renal biopsy was indicated in 0.14%. By eliminating the costs associated with assessment of the results by the Ikeda Medical Association, and by directly contracting with the testing company, the expenses paid by Ikeda City for the system itself decreased from 2,508,619 yen to 966,157 yen.

**Conclusions:**

Incorporating the urinary protein/creatinine ratio into the school urinary screening system in the Ikeda City School System and clarifying standards for specialist referral has enabled restructuring of the system so that is efficient and its effectiveness can be assessed.

## Background

Annual urinary screening has been conducted in the Ikeda City School System based on revisions in the Enforcement Ordinance and Enforcement Regulations of the School Health Law in 1973. The school urinary screening results are evaluated by general physicians who are members of the local medical association. However, standards for when to refer for specialty consultation have not been clear, the number of children who have undergone renal biopsy and aggressive treatment such as steroid therapy is unknown, and it has not been possible to assess effectiveness.

The objective of the school urinary screening has recently been focused on the detection of chronic nephritic syndrome (CNS). Measurement of the urinary protein/creatinine ratio on secondary testing has been introduced, and a system for referral of all children with proteinuria, defined as a urinary protein/creatinine ratio ≥0.2 g/gCr, for consultation at Ikeda City Hospital, a core medical institution in the region, has been established. The results are now reported. In addition to considering the efficiency of the system, the morbidity rate of CNS was estimated, and the costs of the screening system were considered.

## Methods

Urinary screening by dipstick urinalysis of a first-morning urine sample was performed once or twice in all children attending the Ikeda City School System. In children with either urinary protein (≥1+) or occult blood (≥1+) on the first urinalysis, a second urinalysis of a first-morning urine sample was performed. If dipstick urinalysis at this time showed urinary protein (≥1+), the urinary protein/creatinine ratio was measured using that urine.

In addition, if the second urinalysis showed either urinary protein (≥1+) or occult blood (≥1+), the urinary sediment was then also examined, and the results for the number of white blood cells (cells/hpf), the number of red blood cells (cells/hpf), and the presence or absence of cellular casts were recorded. In children with a urinary protein/creatinine ratio ≥0.2 g/gCr (regardless of the presence or absence of occult blood), their parents/guardians were advised to seek treatment at Ikeda City Hospital. An appointment with the pediatric or nephrology outpatient department at Ikeda City Hospital was arranged by the health education teacher.

If the urinary protein/creatinine ratio was <0.2 g/gCr but either of the two urinalyses showed occult blood (≥1+) or urinary glucose (≥ ±), the test results and a letter recommending consultation with the school doctor or a family practitioner (hereinafter referred to as “school doctor”) were sent to the parents/guardians. In this letter, to exclude acute nephritic syndrome, congenital urological disease, and so on, the doctor was asked to “Please check the child’s blood pressure and whether the child has pretibial edema, and take appropriate blood tests or abdominal ultrasonography as needed.” For children with urinary protein (≤ ±) and a urinary protein/creatinine ratio <0.2 g/gCr on second urinalysis, and with occult blood (≤ ±) and urinary glucose (-) on either of the two urinalyses, only the test results were sent to the parents/guardians. The above protocol is shown in Table 1. Children who were referred to Ikeda City Hospital were examined by a pediatrician or nephrologist.

**Table 1:** School urinary screening protocol and results

**1st Urinalysis**		**2nd Urinalysis**			**Direction**	**Number**
**Protein**	**OB**	**Protein**	**OB**	**Uprot/Ucr ratio**		
-or±	-or±				None	8093
- + or + -or++		Not submitted				11
-or±	+	+	-or±	≥0.2	Ikeda City Hospital	0
-or±	+	+	+	≥0.2		0
+	-or±	+	-or±	≥0.2		11
+	-or±	+	+	≥0.2		0
+	+	+	-or±	≥0.2		0
+	+	+	+	≥0.2		2
-or±	+	+	+	<0.2	School doctor or family practitioner	40
+	-or±	+	+	<0.2		
+	+	+	+	<0.2		
-or±	+	-or±	+			
+	-or±	-or±	+			
+	+	-or±	+			
-or±	+	+	-or±	<0.2		
+	+	+	-or±	<0.2		
-or±	+	-or±	-or±			
+	+	-or±	-or±			
+	-or±	+	-or±	<0.2	Observation	55
+	-or±	-or±	-or±			
Total						8212

OB: occult blood.

Uprot/Ucr ratio: urinary protein/creatinine ratio.

Because most of the children going to elementary school and junior high school go to the municipal schools, it is possible to estimate the morbidity of CNS in elementary and junior high school age children from the numbers of children with suspected CNS found by this system, using the expression below.

The morbidity rate = the number of elementary (junior high) school children with suspected CNS ÷ {number of all children in the municipal elementary (junior high) schools × participation rate for the first urinalysis}.

For children who never underwent a second urinalysis despite repeated recommendations, the calculation was revised from the rate of children with suspected CNS in children who underwent a 2nd urinalysis. The revised method of calculation is mentioned in the results.

The ethical committee of Ikeda City Hospital approved the research proposal and granted permission for the research. Written informed consent was obtained from the patient/guardian prior to taking the first urinalysis.

## Results

The population of Ikeda City as of April 1, 2012 was 103,199. This included 938 4-year-old children, 917 children with an age corresponding to the first grade of elementary school (age 6 years), and 981 children with an age corresponding to the first grade of junior high school (age 12 years). The numbers of these children attending the Ikeda City School System were 192 (20.5%), 829 (90.4%), and 842 (85.8%), respectively. Because children usually continue at the same school where they were first-year students until their graduation, among children living in Ikeda City, about 88% attend a municipal elementary school or a municipal junior high school in Ikeda City. The remainder, 12% of children, goes to private, national, and Osaka prefectural kindergartens and schools. In April 2012, a first urinary screening was recommended in all 8274 children attending the Ikeda City School System, including 388 kindergarten, 5363 elementary school, and 2523 junior high school students. In response to the recommendations, urine samples were submitted from a total of 8212 children (99.3%), including 387 kindergarten, 5349 elementary school, and 2476 junior high school students.

Dipstick urinalysis showed urinary protein (≥1+) or occult blood (≥1+) in 119 (1.4%) of these children (kindergarten 0, elementary school 29, junior high school 90), and a second urinary screening was recommended. Of these students, 108 (90.8%) submitted a sample (elementary school 26, junior high school 82). The sample submission rate for the second urinary screening was 89.7% in elementary school and 91.1% in junior high school students (Table 2). None of the children had urinary glucose (≥ ±). The subsequent protocol is shown in Table 1.

**Table 2:** Children who were subjects of urinary examination

	**Kindergarten**	**Elementary**	**Junior high**	**Total**
All children in municipal kindergartens and schools	388	5363	2523	8274
Participated in 1st urinalysis	387	5349	2476	8212
Recommended for 2nd urinalysis	0	29	90	119
Participated in 2nd urinalysis	0	26	82	108
Urinary protein/creatinine ratio ≥0.2 g/gCr	0	1	12	13

In 13 children who had urinary protein (≥1+) on a second dipstick urinalysis, the urinary protein/creatinine ratio was measured using that urine. The urinary protein/creatinine ratio was ≥0.2 g/gCr in 13 children, including 1 elementary school and 12 junior high school students (Table 3). Of these 13 children, 1 junior high school student had proteinuria/hematuria syndrome since elementary school; CNS was suspected, the child was being seen at another hospital, and renal biopsy was being considered. Follow-up evaluation at Ikeda City Hospital was recommended for the other 12 children. CNS was suspected in 3 children based on proteinuria and hematuria syndrome, and renal biopsy was considered in 1 child. In addition, CNS was suspected in 2 children based on proteinuria syndrome, and renal biopsy was considered in 1 child.

**Table 3:** Children with a urinary protein/creatinine ratio ≥0.2 g/gCr

**School**	**Age (y)**	**Sex**	**Result**	
Junior high	15	M	Chronic nephritic syndrome (renal biopsy considered)	Proteinuria & hematuria
Junior high	15	M		
Junior high	15	M		Proteinuria
Junior high	13	M	Chronic nephritic syndrome (observation)	Proteinuria & hematuria
Junior high	13	F		
Junior high	12	F		Proteinuria
Elementary	11	F	Posture proteinuria	
Junior high	12	M	Urinary tract infection	
Junior high	13	M	Within normal limit	
Junior high	13	M		
Junior high	13	F		
Junior high	12	F		
Junior high	15	M	Did not come for consultation	

CNS was suspected in 6 children (all junior high school students), and this was a newly discovered finding in 5 cases. Of the 6 children with suspected CNS, renal biopsy was judged to be indicated by a specialist in 3 cases. Other diagnoses included postural proteinuria in 1 child (elementary school), a urinary tract infection (resolved) in 1 child, and no abnormalities on evaluation at Ikeda City Hospital in 4 children, who were thus diagnosed as having only a transient abnormality at the time of school urinary screening. One child never received further evaluation despite repeated recommendations.

40 children had urinary protein (≤ ±) or a urinary protein/creatinine ratio <0.2 g/gCr on the second urinary screening, but with occult blood (≥1+) on either of the two urinalyses; consultation with the school doctor was recommended. A total of 55 children had urinary protein (≤ ±) or a urinary protein/creatinine ratio of <0.2 g/gCr on the second urinary screening, and with occult blood (≤ ±) and urinary glucose (-) on both urinalyses; these test results were reported, but no recommendation for further evaluation was made (Table 1).

School urinary screening in the Ikeda City School System in 2012 found no children in kindergarten or elementary school with proteinuria/hematuria syndrome or proteinuria syndrome that was suspicious of CNS. Attendance at kindergarten is not part of compulsory education in Japan. In Ikeda City, children going to municipal kindergartens are in the minority, so the CNS morbidity of children going to kindergartens was not estimated. If there was one elementary school child with CNS, based on the calculation 1÷(5349 × 0.897) = 0.00021, the prevalence of CNS among about the 5300 elementary school children in Ikeda City was estimated at 0.021%. In fact, there was no such child, so the prevalence of CNS among about 5300 elementary school children in Ikeda City was estimated at <0.03%.

Of about 2500 junior high school students, 6 children had suspected CNS based on proteinuria/hematuria syndrome or proteinuria syndrome. This was a new finding in 5 of the 6 children. In particular, 3 of these children might have had progressive CNS based on findings such as proteinuria ≥0.5 g/gCr, and a renal biopsy was considered. If the 1 child who did not have further evaluation despite recommendations for consultation is counted as 0.5 persons with suspected CNS and 0.25 persons in whom renal biopsy is indicated, then based on the calculations 6.5÷(2476 × 0.911) = 0.00288 and 3.25÷(2476 × 0.911) = 0.00144, the prevalence of CNS in about 2500 junior high school students in Ikeda City was 0.29%, and a renal biopsy was judged to be indicated by a specialist in 0.14% of these junior high school students.

In 2011, the cost paid by Ikeda City for school urinary screening, including costs associated with assessment of the results by the Ikeda Medical Association, was 2,508,619 yen, and the cost of urinary examination from the local medical association to the testing company was 1,465,926 yen. However, in 2012, there were no costs associated with assessment of the results, but only the costs of urinary screening. Because of the direct contract with the testing company, even with additional measurement of the urinary protein/creatinine ratio, the cost was decreased to 966,157 yen.

## Discussion

It is desirable to compare the efficiency of the old and new systems. However, in the old system, there were no clear criteria for specialist referral, and it is not possible to know the numbers of children diagnosed with CNS. Therefore, the morbidity rate of CNS was estimated using the new system and compared with previously reported morbidity estimates.

In a report by Utsunomiya et al. on the effectiveness of school urinary screening in elementary and junior high school students in Yonago City [1], of 688 children requiring consultation for further evaluation based on renal screening at elementary and junior high schools, 29 (4.2%) underwent renal biopsy; 2 had normal findings, and 27 (3.9%) had a histopathologic diagnosis such as IgA nephropathy. On school urinary screening in the Ikeda City School System, 108 children had a second urinalysis, and in 3 (2.8%) of these children, a renal biopsy was indicated. Thus, the frequency of an indication for renal biopsy in children with abnormal urinary screening results is about the same in Yonago City and Ikeda City.

In a report by Shoji et al. on urinary screening at Osaka Prefectural schools using the urinary protein/creatinine ratio mainly in senior high school students [2], the incidence in 2007 of newly diagnosed chronic glomerulonephritis was 7.6 per 100,000 persons (0.0076%). This is a fairly low incidence, only about 1/18 of the estimated 0.14% prevalence of junior high school children in Ikeda City in whom renal biopsy was indicated. The reasons for this difference may be: new onset cases of chronic glomerulonephritis from 2005 were ascertained in Osaka Prefectural schools, and children with onset by 2006 were not included; only children who were diagnosed with chronic glomerulonephritis based on renal biopsy were counted; and differences between children in senior high school and children in junior high school and lower grades.

In Korea, school children have undergone urine screening tests since 1998. Between 1999 and 2008, a total of 47,047,545 school children, including elementary school children (6–11 years old), middle school children (12–14 years old), and high school children (15–17 years old) participated in a mass school urine screening program. Through this process, 5114 children (0.010%) were referred to pediatric nephrologists, all members of the Korean Study Group of School Urinalysis Screening, at seven hospitals nationwide. Percutaneous renal biopsies were performed on 1478 children (28.9% of referred children), and chronic glomerulonephritis was detected in 25% of referred children [3]. Due to the difference in referral criteria, it is difficult to compare their results with those of the present study.

A cut-off value of 0.2 g/gCr for the urinary protein/creatinine ratio in children aged ≥2 years has previously been reported [4,5], but use of the urinary protein/creatinine ratio in screening systems such as school urinary screening has seldom been reported. In the report from Osaka Prefectural schools, the urinary protein/creatinine ratio was introduced for school urinary screening starting in 2006, and the number of children in whom further detailed evaluation was deemed necessary decreased from 2436 children in 2005 to 268 children in 2007. However, the number of children newly diagnosed with chronic glomerulonephritis did not decrease from 2005 (7 children) to 2007 (9 children) [2]. Thus, the report concluded that the burden on children themselves, their parents/guardians, the schools, and medical institutions was reduced, but without a decrease in diagnostic efficiency. In the case of the Ikeda City School System, although diagnostic efficiency could not be compared, because the number of cases of CNS up to 2011 could not be ascertained, the costs of the urinary screening system could be reduced.

In Japan, IgA nephropathy accounts for ≥30% of cases of chronic glomerulonephritis in adults and ≥20% of cases in children [6]. We also consider early detection of IgA nephropathy to be an important objective of school urinary screening. It has been reported that about 62% of IgA nephropathy in children in Japan is discovered based on microscopic hematuria and/or asymptomatic proteinuria [7], and school urinary screening has played a contributory role. In the “Clinical guidelines for Immunoglobulin A (IgA) nephropathy in Japan, third version,” persistent microscopic hematuria is a necessary finding, and intermittent or persistent proteinuria is a frequent finding, but a small number of patients may have IgA nephropathy without proteinuria [6]. By recommending specialty referral only for children with a urinary protein/creatinine ratio ≥0.2 g/gCr, it may not be possible to diagnose IgA nephropathy without proteinuria. However, the “Clinical guidelines for Immunoglobulin A (IgA) nephropathy in Japan, third version” state that the renal prognosis in clinical severity C-Grade I (urinary protein <0.5 g/day) is relatively good. Furthermore, renal biopsy in children positive only for hematuria but with a urinary protein/creatinine ratio <0.2 g/gCr increases medical costs. In addition, in Japan, which has led the world in conducting school urinary screening nationwide for about 30 years, microscopic hematuria in elementary and junior high school children is almost always associated with a good prognosis [8]. Therefore, in children with a urinary protein/creatinine ratio <0.2 g/gCr, a recommendation for specialty referral is probably not necessary.

In children not seen by specialists, the possibility for presenting with symptoms of CNS in the future exists. This is clear from the higher morbidity of CNS in junior high schools than in elementary schools. To address this issue, annual urinalysis should be continued in schools. In Japan, after graduation from junior high school, 96.6% of children go to senior high school (15–17 years old), where they also undergo urinalyses.

In the new system, the cost of the urinary screening system itself was reduced. However, since referrals were recommended for 53 children (13 children were recommended to see specialists, and 40 children were recommended to see school doctors), there is a possibility that medical expenses increased. Further consideration of ways to decrease recommendations for referrals for children who do not need them is required.

## Conclusions

In conclusion, incorporating the urinary protein/creatinine ratio into the school urinary screening system in the Ikeda City School System and clarifying standards for referral to specialty consultation has enabled restructuring of the system so that it is efficient and its effectiveness can be assessed.

## Competing interests

The authors have no conflicts of interest to disclose. There is nothing to declare with respect to this article.

## Authors’ contributions

NK drafted the manuscript. NK, KH, SY, and IM examined the children at Ikeda City Hospital. NK, TF, KS, SM, TK, and TU designed the study. NK, SH, and SA contributed to data analysis. MI contributed as a representative of school doctors and family practitioners in Ikeda City. SH and KS contributed as representatives of the Ikeda City Board of Education. MY and SA contributed as representatives of health education teachers in Ikeda City. All authors read and approved the final manuscript.
